# OSR1 and SPAK cooperatively modulate Sertoli cell support of mouse spermatogenesis

**DOI:** 10.1038/srep37205

**Published:** 2016-11-17

**Authors:** Yung-Liang Liu, Sung-Sen Yang, Shyi-Jou Chen, Yu-Chun Lin, Chin-Chen Chu, Hsin-Hui Huang, Fung-Wei Chang, Mu-Hsien Yu, Shih-Hua Lin, Gwo-Jang Wu, Huey-Kang Sytwu

**Affiliations:** 1 Graduate Institute of Medical Sciences, National Defense Medical Center, Taipei 114, Taiwan; 2 Department of Obstetrics and Gynecology, Tri-Service General Hospital, National Defense Medical Center, Taipei 114, Taiwan; 3 Department of Obstetrics and Gynecology, Tri-Service General Hospital Penghu Branch, Penghu 880, Taiwan; 4 Division of Nephrology, Department of Medicine, Tri-Service General Hospital, National Defense Medical Center, Taipei 114, Taiwan; 5 Department and Graduate Institute of Microbiology and Immunology, National Defense Medical Center, Taipei 114, Taiwan; 6 Department of Pediatrics, Tri-Service General Hospital, National Defense Medical Center, Taipei 114, Taiwan; 7 Department of Pathology, Tri-Service General Hospital, National Defense Medical Center, Taipei 114, Taiwan; 8 Department of Anesthesiology, Chi Mei Medical Center, Tainan, Taiwan; 9 Department of Recreation and Health-Care Management, Chia Nan University of Pharmacy and Science, Tainan, Taiwan; 10 Graduate Institute of Life Sciences, National Defense Medical Center, Taipei 114, Taiwan

## Abstract

We investigated the role of oxidative stress-responsive kinase-1 (OSR1) and STE20 (sterile 20)/SPS1-related proline/alanine-rich kinase (SPAK), upstream regulators of the Na^+^-K^+^-2Cl^−^ cotransporter (NKCC1)—essential for spermatogenesis—in mouse models of male fertility. Global OSR1^+/−^ gene mutations, but not global SPAK^−/−^ or Sertoli cell (SC)-specific OSR1 gene knockout (SC-OSR1^−/−^), cause subfertility with impaired sperm function and are associated with reduced abundance of phosphorylated (p)-NKCC1 but increased p-SPAK expression in testicular tissue and spermatozoa. To dissect further in a SC-specific manner the compensatory effect of OSR1 and SPAK in male fertility, we generated SC-OSR1^−/−^ and SPAK^−/−^ double knockout (DKO) male mice. These are infertile with defective spermatogenesis, presenting a SC-only-like syndrome. Disrupted meiotic progression and increased germ cell apoptosis occurred in the first wave of spermatogenesis. The abundance of total and p-NKCC1 was significantly decreased in the testicular tissues of DKO mice. These results indicate that OSR1 and SPAK cooperatively regulate NKCC1-dependent spermatogenesis in a SC-restricted manner.

Recent studies have indicated that Na^+^-K^+^-2Cl^−^ cotransporters (NKCCs), which comprise the ubiquitous NKCC1 and the renal-specific NKCC2, play important roles in the regulation of blood pressure (BP) and cell volume^1^,^2^,^3^,^4^. Male reproductive tissues, such as Sertoli cells (SCs), pachytene spermatocytes (PSs), round spermatids (RSs), and mature spermatozoa, express NKCC1 abundantly^5^,^6^. Accumulating evidence has shown that NKCC1 is critical for male fertility. Thus, male NKCC1 knockout (KO) mice are infertile because of defective spermatogenesis and abnormal acrosomal development^5^. Moreover, NKCC1 is likely to be responsible for the inward translocation of Cl^−^ in spermatozoa, and these NKCC-dependent Cl^−^ currents subsequently contribute to the zona pellucida-induced acrosome reaction (AR)^6^. Consistent with the important role for this cotransporter in male fertility, NKCC1 inhibitors have been shown to display inhibitory effects on *in vitro* fertilization (IVF)^6^. However, the molecular mechanisms involved in modulating NKCC1 in male fertility are still not completely understood.

Oxidative stress-responsive kinase-1 (OSR1) and sterile 20 (STE20)/SPS1-related proline/alanine-rich kinase (SPAK) are two mammalian members of the STE20 superfamily of mitogen-activated protein kinase-like protein kinases^7^,^8^. OSR1 and SPAK act as upstream regulators of NKCCs by utilizing their conserved C-terminus (CCT) domains to interact with the RFXV/I motif(s) in the N-terminus regions of their NKCC substrates^9^. Phosphorylation of threonine residues of NKCC1/2 (T206/96, T211/101, and T224/114 in the mouse) on their N-terminal conserved domain by OSR1 and SPAK enhances their function^9^,^10^,^11^. Mouse models with global SPAK KO, a heterozygous OSR1 mutation, or kidney-specific OSR1 KO reveal that these two kinases can regulate ion homeostasis and BP through phosphorylating and activating NKCCs^12^,^13^. These findings indicate that both OSR1 and SPAK are important regulators of NKCC *in vivo*. Given that NKCC1 is critical for spermatogenesis *in vivo*^5^ and that OSR1 and SPAK are upstream regulators of NKCC^12^,^13^, testing how OSR1 and SPAK might regulate NKCC1 could be important in revealing new aspects of the genetic control of male fertility.

Germinal centre kinase (GCK)-3, an orthologue of OSR1 and SPAK in *Caenorhabditis elegans (C. elegans*), interacts with CLH3, a ClC-type chloride channel, and mediates fertility^14^,^15^. *C. elegans* with a *gck-3* deletion produce fewer sperm cells from the spermathecae, resulting in decreased fertilization rates. This phenotype suggests that *gck-3* is critical for fertility in such hermaphrodite animals^15^. However, little is known about the role of OSR1 and SPAK in male fertility and sperm function in mammals.

In this study, we first analysed global OSR1^+/−^ and SPAK^−/−^ KO mice to elucidate the physiological role of these factors in male fertility. Mice with a global OSR1^+/−^ mutation, but not a global SPAK deletion, showed subfertility. To discriminate whether OSR1^+/−^ mutation-mediated subfertility is regulated in a genital tissue-specific manner, we generated Sertoli cell (SC)-specific OSR1 gene KO mice (SC-OSR1^−/−^). Unexpectedly, the time to first litter of male SC-OSR1^−/−^ mice was similar to that of control mice. To dissect further the compensatory effects of OSR1 and SPAK on regulation of male fertility in a SC-specific manner, we generated SC-OSR1^−/−^ and SPAK^−/−^ double knockout (DKO) male mice. These DKO mice displayed infertility with increased germ cell apoptosis and defective spermatogenesis, manifesting as a SC-only-like syndrome. We found that the abundance of total and p-NKCC1 was significantly decreased in the testicular tissues of DKO mice compared with those of control mice. This study provides *in vivo* evidence that OSR1 cooperates with SPAK in SCs to regulate spermatogenesis via the activation of NKCC1.

## Results

### Expression of OSR1 and SPAK in mouse testis and Sertoli cells

**Figure 1:**
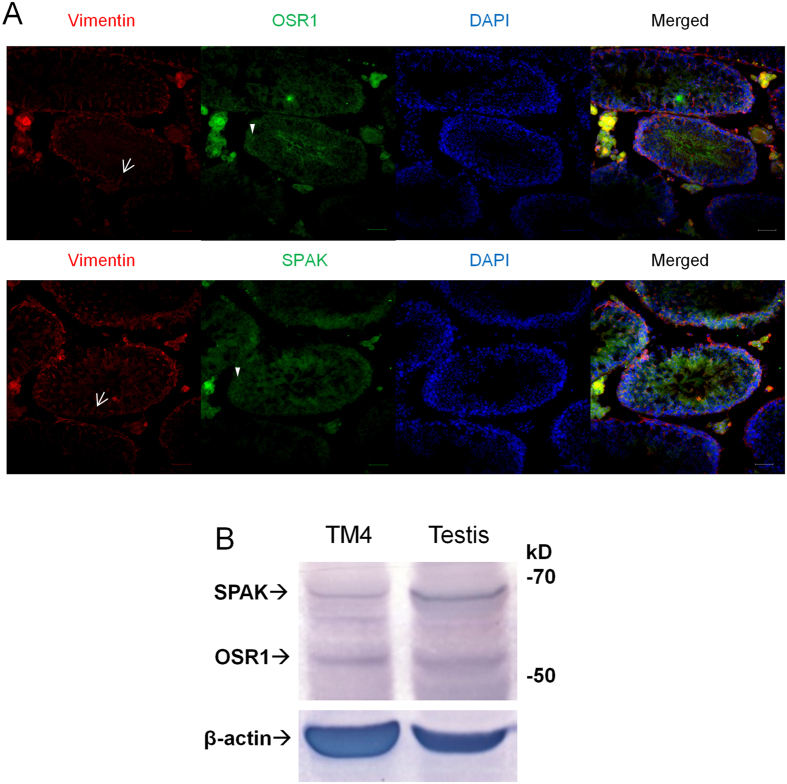
OSR1 and SPAK expression in Sertoli cells. (**A**) OSR1 (green; upper panel) expression in adult testes at 5 months of age. Vimentin (red) was expressed in the SC cytoplasm, and characteristic apical filament projections (arrow) were observed in adult testes. Double immunostaining for OSR1 and vimentin revealed that OSR1 was colocalized with vimentin in SCs. OSR1 was also expressed in germ cells (arrowhead). SPAK (green; lower panel) expression in adult testes at 5 months. The vimentin (red) was expressed in the SC cytoplasm, and characteristic apical filament projections (arrow) were observed in adult testes. Double immunostaining of SPAK and vimentin revealed that SPAK was colocalized with vimentin in SCs. SPAK was also expressed in germ cells (arrowhead). Nuclei were counterstained with DAPI (blue). Scale bar: 50 μm. (**B**) Expression of OSR1 and SPAK protein in TM4 Sertoli cell line. Extracts of TM4 Sertoli cell line and testicular tissues of adult WT mice, used as a positive control, were analysed by SDS-PAGE and western blotting with the indicated Abs. β-Actin was used as a loading control. Data are representative of three independent experiments.

To determine whether OSR1 and SPAK play a role in male fertility, we analysed OSR1 and SPAK expression in adult mouse testes. Co-immunostaining for OSR1 or SPAK with vimentin (a marker of SC microtubules)^16^ revealed that OSR1 and SPAK were expressed in SCs (Fig. 1A). In addition, we used TM4 cells (a mouse Sertoli cell line) to analyse OSR1 and SPAK expression in SCs by western blotting. Protein expression of these two kinases was detected in TM4 cells (Fig. 1B). These data confirmed that OSR1 and SPAK are both expressed in SCs.

### Phenotypic assessment in global OSR1^+/−^ mice

**Figure 2:**
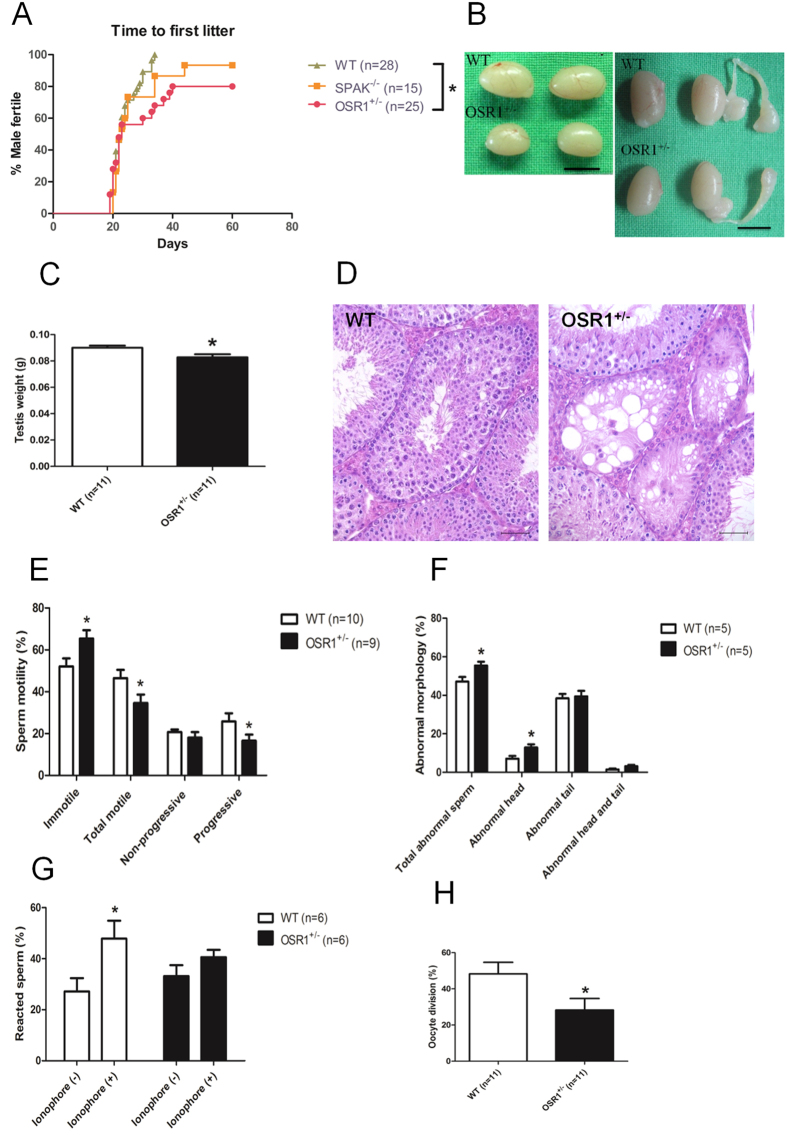
Fertility, phenotypic and sperm assessment. (**A**) Time to first litter. One male was crossed with one WT female mouse. Fertility was confirmed by the number of males (percent) who were able to sire their first litter relative to time (days). Significance was evaluated by log-rank test (*p < 0.05, OSR1^+/−^ vs. WT). (**B**) Testis size (left panel: without epididymis; right panel: with epididymis). Scale bar: 50 mm. (**C**) Testis weight. OSR1^+/−^ testes were smaller than those of WT mice. (**D**) Histological analysis of testis sections. Cystic-like degeneration of seminiferous tubules was noted in adult OSR1^+/−^ mice. Scale bar: 50 μm. (**E**) Sperm motility. The sperm motility was graded as progressive motility, non-progressive motility, or immotility. The total of motile spermatozoa included progress plus non-progressive motility. There were significantly fewer motile and progressing sperm in OSR1^+/−^ mice than in WT mice. (**F**) Sperm morphology. Abnormal spermatozoa were classified into spermatozoa with abnormal head only, spermatozoa with abnormal tail only, and spermatozoa with abnormal head and tail. The percentage of spermatozoa with abnormal morphology was greater in OSR1^+/−^ mice than in WT mice, especially in terms of head abnormalities. (**G**) ARs. After one h of capacitation, calcium ionophore (A23187; 10 μM) was added, and spermatozoa were incubated for an additional 30 min. PNA was used to assess the acrosomal status. In all experiments, reacted spermatozoa were classified as those with a spontaneous AR (without ionophore) and those with an ionophore-induced AR. The percentage of ARs in OSR1^+/−^ spermatozoa following A23187 stimulation was not increased significantly compared with that in stimulated WT spermatozoa, implying an inability of partially OSR1-deficient spermatozoa to undergo an ionophore-induced AR. (**H**) IVF. Spermatozoa (3 × 10^5^) incubated with 20–30 oocytes in insemination drops were used in IVF assays. The capacity of OSR1^+/−^ spermatozoa to fertilize oocytes after 24 h incubation was significantly lower than that of WT spermatozoa. All values in C and E–H are given as the mean ± SEM. *p < 0.05, **p < 0.01 by two-tailed Student’s unpaired *t* test.

Male NKCC1 KO mice are infertile because of failure in spermatogenesis^5^. OSR1 and SPAK are two upstream phosphorylators of NKCC1; however, their roles in male fertility have not been studied thoroughly. Although global OSR1^−/−^ mice die during embryogenesis, the growth of OSR1^+/−^ mice is normal^13^. In contrast, global SPAK^−/−^ mice grow normally and are indistinguishable from control littermates (wild-type, WT) in appearance, behaviour, and fertility^12^. To investigate the potential effect of OSR1 and SPAK, we first compared the time to first litter and litter sizes between male OSR1^+/−^, SPAK^−/−^, and WT mice. Mating male OSR1^+/−^ mice with fertile WT female mice resulted in a significant reduction in the proportion with a time to first litter within the 60 d mating period compared with control mice, indicating that attenuation of OSR1 resulted in reduced male fertility (Fig. 2A). In contrast, the time to first litter of male SPAK^−/−^ mice was indistinguishable from that of their control counterparts (Fig. 2A), suggesting a minimal effect of SPAK deficiency on male fertility. However, the mean number of offspring per litter did not differ between the three groups (Fig. S1A). Because the proportion of male OSR1^+/−^ mice that sired a litter was decreased significantly, we used these mice, rather than SPAK^−/−^ mice, to dissect further the effects of attenuated levels of OSR1 on male fertility.

To investigate whether attenuated OSR1 would affect male fertility by modulating the development of reproductive tissues, we first compared the gross appearances and histology of these tissues between OSR1^+/−^ and WT mice at 8–10 weeks of age. Male OSR1^+/−^ mice displayed normal external genitalia, and their body weights were similar to those of age-matched WT mice (Fig. S1B). The testes of adult OSR1^+/−^ mice showed a modest but significant reduction in size compared with those of WT mice (Fig. 2B,C). The epididymal weights of adult OSR1^+/−^ mice were similar to those of WT mice (Fig. S1C). We then analysed histological sections of testicular and epididymal tissues and measured serum testosterone levels. The testicular histology of three of six OSR1^+/−^ mice revealed a small proportion of cystic changes in the seminiferous tubules (Fig. 2D). These cystic changes were not observed in WT mice (Fig. 2D). There were no significant histological differences in the epididymal tissues of WT and OSR1^+/−^ mice (Fig. S1D). The serum testosterone levels of adult OSR1^+/−^ mice were lower than those of WT mice, but this difference was not significant (Fig. S1E). Collectively, our results revealed that attenuated OSR1 expression reduced testicular size, which might arise partly from cystic changes in the seminiferous tubules of OSR1^+/−^ mice.

To investigate whether attenuated OSR1 would affect sperm count and motility, we analysed epididymal sperm extracts. The cauda epididymal sperm count was similar in WT and OSR1^+/−^ males (Fig. S1F), indicating that attenuated OSR1 had a minimal effect on the total numbers of spermatozoa in this tissue. However, the percentage of motile spermatozoa was decreased in OSR1^+/−^ mice compared with WT mice, especially for progressive motility (Fig. 2E). Moreover, the percentage of spermatozoa with abnormal morphology was increased in OSR1^+/−^ mice compared with that in WT mice, especially in terms of head abnormalities (Fig. 2F). Collectively, our results suggest that attenuated OSR1 expression might directly or indirectly cause these changes in epididymal spermatozoa.

### Effect of partial OSR1 deficiency on the AR and IVF capacity of spermatozoa

The AR is one of the main steps in fertilization and is a stable measure of sperm function. The calcium ionophore-induced AR is a useful indicator of fertility potential for intrauterine insemination and in conventional IVF^17^. To investigate whether partial OSR1 deficiency would affect this, we studied the calcium ionophore (A23187)-induced AR (Fig. 2G). Reacted spermatozoa were classified into those with a spontaneous AR (without ionophore) and those with an ionophore-induced AR. The spontaneous AR in WT and OSR1^+/−^ spermatozoa was indistinguishable, indicating that a partial deficiency of OSR1 was not sufficient to affect spontaneous ARs. However, the percentage of ARs following A23187 stimulation of OSR1^+/−^ spermatozoa, unlike that in stimulated WT spermatozoa, was not increased significantly, implying an inability of partially OSR1-deficient spermatozoa to undergo an ionophore-induced AR. To determine further the fertilization function of OSR1^+/−^ spermatozoa, we performed IVF assays. The capacity of OSR1^+/−^ spermatozoa to fertilize oocytes after 24 h incubation was significantly lower than that of WT spermatozoa (Fig. 2H), suggesting that a partial deficiency of OSR1 causes a defect in sperm IVF potential.

### Expression of OSR1, SPAK, and NKCC1 and their phosphorylation status in testicular tissues and mature spermatozoa

**Figure 3:**
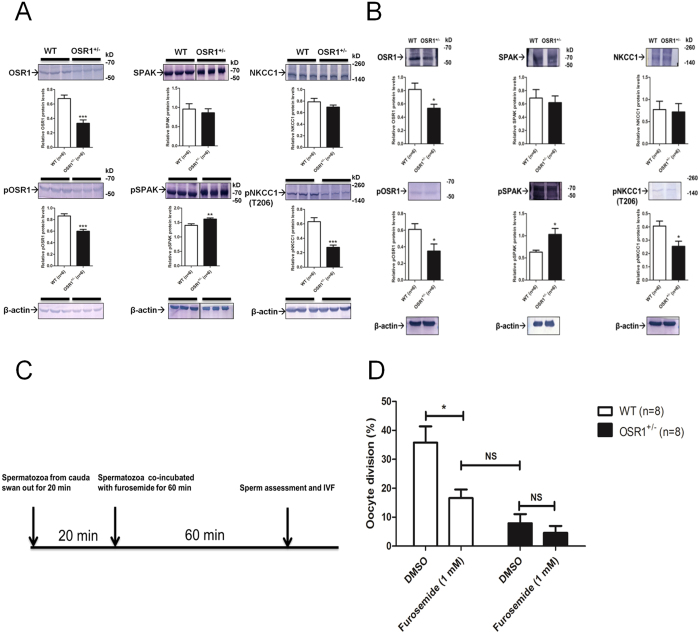
Expression of OSR1, SPAK, and NKCC1 and their phosphorylation status in testicular tissues and mature spermatozoa, and effect of an NKCC1 inhibitor on fertilization. (**A**) Semi-quantitative IB (upper) and densitometry (lower) of total and p-OSR1, total and p-SPAK, and total and p-NKCC1 (T206) in testicular tissues of WT and OSR1^+/−^ mice. Data are representative of two independent experiments. (**B**) Semi-quantitative IB (upper) and densitometry (lower) of total and p-OSR1, total and p-SPAK, and total and p-NKCC1 (T206) in spermatozoa of WT and OSR1^+/−^ mice. Data are representative of at least three experiments in spermatozoa. Data are presented as the mean ± SEM. Significance was evaluated by a two-tailed Student’s unpaired t test. *p < 0.05, **p < 0.01 and ***p < 0.0001 vs. WT. (**C**) Schematic diagram of the experiment. Spermatozoa were isolated from caudas of WT or OSR1^+/−^ mice and swam out for 20 min. Then, spermatozoa were co-incubated with furosemide for 60 min in capacitation medium. After washing, sperm assessment and IVF were performed. (**D**) Furosemide-inhibited IVF. Eggs were inseminated with capacitated spermatozoa in the presence of furosemide. Data are presented as mean ± SEM. *p < 0.05 by two-tailed Student’s unpaired *t* test.

Because NKCC1 is phosphorylated and activated by OSR1/SPAK^18^,^19^, and this cotransporter activity is known to play an important role in the regulation of male spermatogenesis^5^, we examined whether the OSR1/SPAK-NKCC1 pathway was involved in the subfertility of these OSR1^+/−^ mice. The protein expression levels of total and p-OSR1/SPAK/NKCC1 in the testicular tissues and mature spermatozoa of OSR1^+/−^ mice were examined. We found that the expression of functional p-NKCC1 (T206) was significantly decreased in OSR1^+/−^ mice compared with WT mice, in parallel with the reductions in total OSR1 and p-OSR1 in the testes (Fig. 3A) and in mature spermatozoa (Fig. 3B). In contrast, the p-SPAK level was significantly increased with unchanged total SPAK in the testes (Fig. 3A) and mature spermatozoa (Fig. 3B) of OSR1^+/−^ mice compared with that of WT mice. Immunofluorescence (IF) staining revealed that p-NKCC1 (T206) was mainly located in the acrosomal region of the head in WT spermatozoa (panel A) and the signal was markedly reduced in OSR1^+/−^ spermatozoa (panel B) (Fig. S2). Moreover, the overall fluorescence intensity in OSR1-deficient spermatozoa was much lower than that in WT spermatozoa (Fig. S2), consistent with the results observed in western blot analysis. Collectively, these findings suggest that OSR1 haploinsufficiency may cause decreased p-NKCC1 expression in testes and mature spermatozoa, which contributes to male subfertility. Although a possible compensation caused by impaired OSR1 function might have increased the level of p-SPAK, the overall p-NKCC1 level was still reduced, implying that OSR1 principally phosphorylates NKCC1 in these tissues.

### Effect of an NKCC inhibitor on fertilization

To investigate whether OSR1 would affect sperm function directly, we isolated mature spermatozoa from the caudal epididymides of male OSR1^+/−^ and WT mice and incubated these cells with a pharmacological inhibitor. We chose furosemide, an NKCC1 inhibitor, because specific inhibitors of OSR1 were unavailable. A schematic diagram of the experimental approach is presented in Fig. 3C. Sperm cells incubated with furosemide for 1 h were washed, and IVF was performed. The IVF rate of WT spermatozoa treated with furosemide was significantly decreased to a level similar to that of OSR1^+/−^ spermatozoa incubated in control medium (Fig. 3D), indicating that the impaired fertilizing capacity of OSR1^+/−^ spermatozoa was regulated in an OSR1–NKCC1 axis-dependent manner.

### Generation of tissue-specific OSR1 KO mice

The expression of OSR1 and SPAK was detected in testicular tissues, including SCs and germ cells (Fig. 1A). Global SPAK KO mice displayed normal male fertility. Global OSR1^+/−^ mice manifested male subfertility with incomplete phenotypic penetration. It was therefore of interest to dissect OSR1 function in SCs and germ cells, respectively, by generating germ cell- and SC-specific OSR1 KO mice. Although in this study we were unable to generate germ cell-specific OSR1 KO mice, we successfully generated SC-specific OSR1 KO mice (SC-OSR1^−/−^) by using SC-specific anti-Müllerian hormone (AMH) promoter-driven recombinase transgenic mice^20^,^21^,^22^,^23^.

The expression of total OSR1 was attenuated in the testes of SC-OSR1^−/−^ mice compared with those of control mice (Fig. S3A). Because the AMH-driven Cre has been demonstrated to excise effectively the floxed sequence in these transgenic mice, the presence of a certain amount of OSR1 detected in western blots could be contributed by testicular cells other than SCs isolated from these SC-OSR1^−/−^ mice. To investigate whether a specific ablation of OSR1 in SCs would affect male fertility, we assessed the fertility of SC-OSR1^−/−^ mice. Despite the deletion of the OSR1 gene in SCs, the time to first litter (Fig. S3B) and the average litter size (Fig. S3C) were similar to those of control mice, suggesting a minimal effect of OSR1 deficiency in SCs in the regulation of male fertility. In combination with our data showing that the level of p-SPAK was significantly increased in the testes of OSR1^+/−^ mice (Fig. 3A), these results imply that other pathway(s), such as the parallel kinase SPAK, can compensate physiologically for the loss of OSR1 kinase activity in SCs.

### Upregulation of p-OSR1 in the absence of SPAK in testicular tissues

To investigate whether OSR1 can compensate for the loss of SPAK in testicular tissues, we examined the abundance of total and phosphorylated OSR1 and SPAK proteins in the testicular tissues of SPAK^−/−^ mice. We found that in the testes of SPAK^−/−^ mice, the abundance of functional p-OSR1 was significantly increased with unchanged total OSR1 (right column, Fig. S4) compared with WT mice. The presence of total and p-SPAK in the testes of SPAK^−/−^ mice was barely detectable, demonstrating a conventional KO phenotype for SPAK in these mice (left column, Fig. S4). This result implied that OSR1 can compensate for the loss of SPAK activity in testicular tissues, which may contribute to the normal male fertility observed in SPAK^−/−^ mice. Hence, we hypothesized that OSR1 or SPAK can compensate physiologically for the loss of SPAK or OSR1 activity in SCs.

### Generation of SC-OSR1^−/−^ and SPAK^−/−^ DKO mice and assessment of their fertility

SPAK KO mice displayed normal male fertility, indicating that a deficiency of SPAK in either SCs or in germ cells did not affect male fertility. Moreover, SC-OSR1^−/−^ mice had similar fertility outcomes to control mice, suggesting that a deficiency of OSR1 in SCs does not influence this. Based on a report that OSR1 and SPAK participate equally in activating NKCC1 in dorsal root ganglion neurons^24^, and our findings that increased p-SPAK was observed in testicular tissues from OSR1^+/−^ mice (Fig. 3A) and that p-OSR1 was upregulated in testicular tissues from SPAK^−/−^ mice (Fig. S4), we hypothesized that these two kinases might cooperate to phosphorylate NKCC1 in SCs to regulate male fertility. To address this issue, we generated double knockout (DKO) mice from SC-OSR1^−/−^ and global SPAK knockout mice that had both kinases ablated in SCs. We verified that OSR1 and SPAK were ablated in the SCs of the DKO mice using polymerase chain reaction (PCR) (Fig. S5), IF staining, and reverse transcription-quantitative PCR (RT-qPCR) (Fig. S6). We verified that the allele lacking exons 9 and 10 (OSR1 [Δexon 9, 10]) after the AMH Cre recombination event (307 bp) was detected in testes but not in other tissues (Fig. S5). IF staining revealed that the signals for OSR1 and SPAK were markedly reduced in the seminiferous tubules of DKO mice compared with controls (Fig. S6A,B). Both *Oxsr1* and *Spak* transcripts were significantly downregulated in testicular tissues from DKO mice compared with controls (Fig. S6C). Collectively, these findings supported that both OSR1 and SPAK were ablated in the SCs of DKO mice. We then evaluated the male fertility function of the DKO mice, and found that they were completely infertile.

### Inactivation of OSR1 and SPAK in SCs resulted in germ cell loss and a SC-only-like appearance

**Figure 4:**
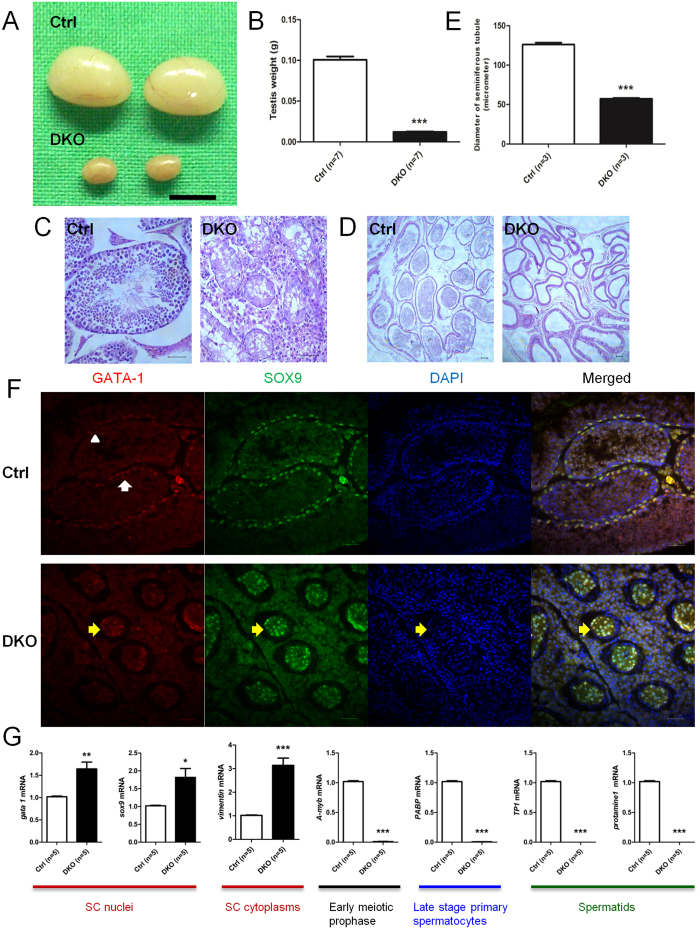
Fertility and phenotypic assessment in DKO mice. (**A**) Testis size. Scale bar: 50 mm. (**B**) Testis weight. DKO testes were smaller than those of control mice. (**C**) Histological analysis of adult testes. Developing germ cells at all stages were observed in control mice, but smaller seminiferous tubules with Sertoli cell-only-like syndrome occurred in DKO males. Scale bar: 50 μm. (**D**) Histological analysis of adult cauda epididymidis. Spermatozoa could only be detected in control mice but not in DKO males. Scale bar: 50 μm. (**E**) The diameter of seminiferous tubules of adult control and DKO mice. (**F**) DKO mouse testes manifested a SC-only-like appearance. SCs were stained with GATA-1 (red) and SOX9 (green). Two types of seminiferous tubules existed in transverse sections of adult control mouse testes at 5 months: one containing stronger (upper panel, arrow) and one containing weak signals (upper panel, arrowhead) for GATA-1-positive SCs; this represents spermatogenic cycle-specific expression. Double immunostaining for GATA-1 and SOX9 revealed that GATA-1 was colocalized with SOX9 in the nuclei of SC in adult control mouse testes (upper panel). The diameters of seminiferous tubules (lower panel, yellow arrow) of DKO testes at 5 months were smaller than those of control mice. In the seminiferous tubules of DKO mice, almost all nuclei were SOX9 and GATA-1-positive (lower panel, yellow arrow), giving a SC-only-like appearance. Nuclei were counterstained with DAPI (blue). Scale bar: 50 μm. (**G**) mRNA expression levels of the indicated genes in testicular tissues of control and DKO mice at 5 months of age as determined by RT-qPCR normalized to *Rps2* expression. All values in (**B**,**E**,**G**) are given as the mean ± SEM. ***p < 0.0001 by two-tailed Student’s unpaired *t* test.

Analysis of the testes of DKO mice revealed a significant reduction in size to about 10% of that in control mice (Fig. 4A,B). The testicular histology of DKO mice at 5 months of age showed smaller tubules with a SC-only-like appearance (Fig. 4C,E), and no spermatozoa were detected in sections of the epididymides of DKO males (Fig. 4D). GATA-1, a member of the GATA transcription factor family, is expressed exclusively by SCs of the testis^25^,^26^,^27^,^28^,^29^. It has been suggested that its expression is negatively regulated by mature germ cells and that it shows developmental stage- and spermatogenic cycle-specific expression in SCs^27^. To identify unambiguously the lineage of the cells in the seminiferous tubules of DKO testes, we examined the expression of GATA-1 and SOX9, which is a member of the SOX [Sry-related high-mobility group (HMG) box] family and a SC nuclear marker. In control adult mouse testes, the expression of GATA-1 and SOX9 was restricted to the SC lineage (Fig. 4F). In the DKO mice, the distribution of GATA-1- and SOX9-positive cells occupied almost all of the atrophic seminiferous tubules (Fig. 4F), supporting the notion that the testes of DKO mouse were almost free of germ cells. We also found significant near-twofold higher levels of mRNA for both *Gata-1* and *Sox9* and significant threefold higher levels of mRNA for *vimentin* in the testicular tissues of DKO mice compared with control mice (Fig. 4G). This finding provided further evidence to support the notion that there is an increased proportion of SCs in DKO mice. We next examined the expression of genes for selected differentiation markers (A-Myb, PABP, TP1 and protamine1)^30^,^31^,^32^,^33^,^34^,^35^ in the testes of DKO mice. The expression of all these markers was undetectable in the testes of DKO mice at 5 months of age (Fig. 4G). Collectively, these findings indicated that the ablation of both OSR1 and SPAK in SCs resulted in germ cell loss and manifested as a SC-only-like appearance of the testicular tissues of DKO mice.

### Characterization of the first wave of spermatogenesis in DKO mice

**Figure 5:**
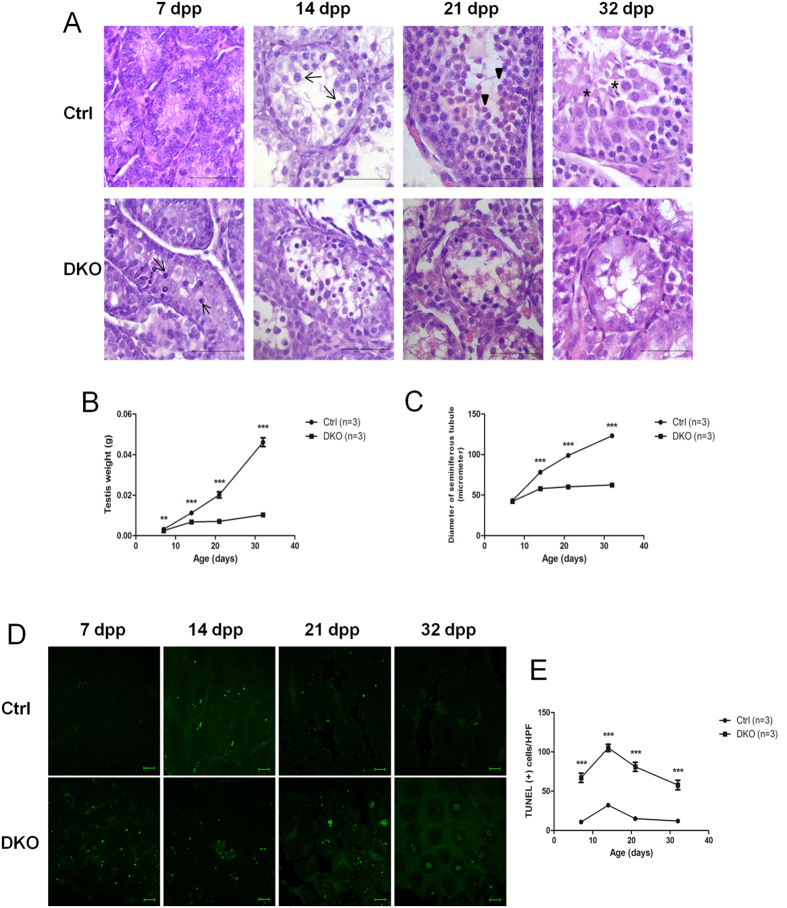
Analysis of the first wave of spermatogenesis and TUNEL assays in testicular tissues of DKO mice. (**A**) Histological analysis of testes at different time points during the first wave of spermatogenesis in control and DKO mice. Scale bar: 50 μm. Testes of control mice collected at 7 dpp contained spermatogenic cells just prior to, or at, the onset of meiosis, whereas this process was retarded, and apoptotic cells (arrows) were noted, in DKO mice. Control testes collected at 14 dpp showed PSs (arrows), but most cells in DKO mice did not reach this stage. Control testes collected at 21 dpp contained RSs (arrowheads), whereas no RSs and decreased numbers of germ cells were observed in DKO mice at this phase. Control testes collected at 32 dpp displayed ESs (asterisks), but most cells in DKO mice were SCs, with ESs barely detectable at this time. (**B**) Testis weight at different time points during the first wave of spermatogenesis in control and DKO mice. (**C**) The diameter of seminiferous tubules at different time points during the first wave of spermatogenesis in control and DKO mice. (**D**) Representative images of TUNEL (green) staining of testis sections at different time points during the first wave of spermatogenesis in control and DKO mice. Scale bar: 50 μm. (**E**) Quantitative analysis of TUNEL staining. At least four high-power fields were selected for analysis of each stain. All values in (**B**,**C**,**E**) are given as the mean ± SEM. **p < 0.01 and ***p < 0.0001 by two-tailed Student’s unpaired *t* test.

To analyse further the cellular events that lead to infertility in DKO mice, we compared the development of control and mutant testes during the first wave of spermatogenesis from 7 days postpartum (dpp) to 32 dpp (Fig. 5A). Testes from control mice at 7 dpp showed spermatogenic cells that had almost reached or were at the onset of meiosis, whereas this process was retarded, and the numbers of apoptotic cells were increased, in DKO mice. PSs appeared in control mice at 14 dpp, but most cells in DKO mice did not reach this stage. RSs were present in the seminiferous tubules in control mice at 21 dpp, whereas no RSs and decreased numbers of germ cells were observed in DKO mice at this phase. Elongated spermatids (ESs) were present in the seminiferous tubules in control mice at 32 dpp; however, most cells in DKO mice at this time were SCs, while ESs were barely present. Furthermore, a gradual increase in testis weight and seminiferous tubule diameter were recorded in control mice during the first wave of spermatogenesis from 7 to 32 dpp, but only slight changes were observed in these parameters in the DKO mice (Fig. 5B,C). Collectively, our results demonstrated that ablation of both OSR1 and SPAK in SCs resulted in early histological abnormalities with retarded meiosis and markedly increased germ cell apoptosis, associated with decreased testis weight and seminiferous tubule diameter during the first wave of spermatogenesis.

### Apoptosis in the first wave of spermatogenesis

To characterize further the apoptotic events that lead to the deletion of most germ cells in DKO testes, we performed terminal deoxynucleotidyl transferase dUTP nick end labelling (TUNEL) assays during the first wave of spermatogenesis from 7 to 32 dpp. Our data revealed that compared with control mice, significant apoptosis appeared in DKO mice at 7 dpp, reached a peak at 14 dpp, and persisted to 32 dpp, indicating that the absence of both OSR1 and SPAK in SCs leads to apoptosis of most germ cells instead of the initiation of meiosis (Fig. 5D,E).

### Expression of NKCC1 and its phosphorylation status in testicular tissues of DKO mice

**Figure 6:**
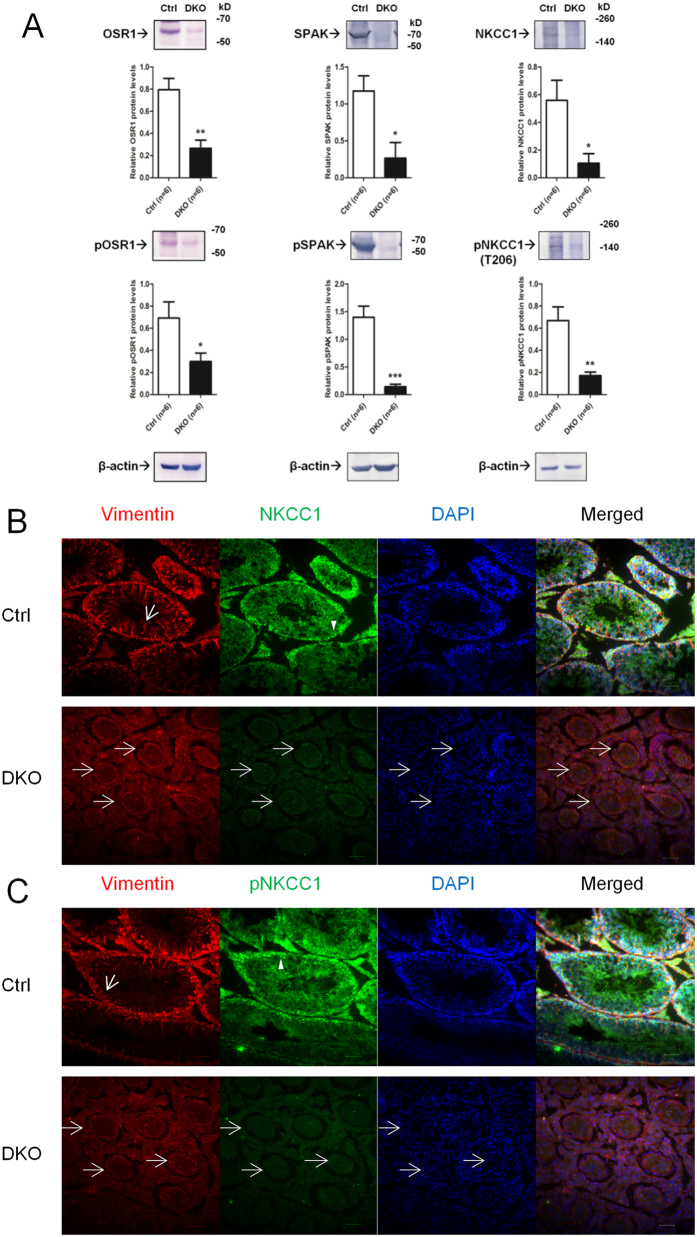
Expression of OSR1, SPAK, and NKCC1 and their phosphorylation status in DKO mice. (**A**) Semi-quantitative IB (upper) and densitometry (lower) for total and p-OSR1, total and p-SPAK, and total and p-NKCC1 (T206) in testicular tissues of adult control and DKO mice. Data are representative of six independent experiments. (**B**) NKCC1 expression in adult testes of control (upper panel, green) and DKO (lower panel, green) mice at 5 months of age. The vimentin (red) was expressed in the SC cytoplasm, and characteristic apical filament projections (arrow) were observed in adult testes of control mice. Double immunostaining for NKCC1 and vimentin revealed that NKCC1 was colocalized with vimentin in SCs. NKCC1 was also expressed in germ cells (arrowhead). The diameters of seminiferous tubules (lower panel, arrow) of DKO testes were smaller than those of control mice. The signals for NKCC1 were markedly reduced in seminiferous tubules (lower panel, arrow) of DKO mice compared with controls. (**C**) p-NKCC1 expression in adult testes of control (upper panel, green) and DKO (lower panel, green) mice at 5 months. Vimentin (red) was expressed in the SC cytoplasm, and characteristic apical filament projections (arrow) were observed in adult testes of control mice. Double immunostaining for p-NKCC1 and vimentin revealed that p-NKCC1 was colocalized with vimentin in SCs. p-NKCC1 was also expressed in germ cells (arrowhead). The diameters of seminiferous tubules (lower panel, arrow) of DKO testes were smaller than those of control mice. The signals for p-NKCC1 were markedly reduced in seminiferous tubules (lower panel, arrow) of DKO mice compared with controls. Nuclei were counterstained with DAPI (blue). Scale bar: 50 μm. All values in A are given as the mean ± SEM. *p < 0.05, **p < 0.01 and ***p < 0.0001 by two-tailed Student’s unpaired *t* test.

We postulated that a physiological compensation between OSR1 and SPAK in SCs might contribute to the regulation of male fertility. To test this hypothesis, we analysed the expression and phosphorylation levels of key molecules involved in the OSR1/SPAK–NKCC1 axis of DKO testicular tissues. In the testes of DKO mice, the signals for total and p-SPAK were barely detectable, demonstrating the lack of SPAK expression in these mice (middle column, Fig. 6A). The amounts of total and p-OSR1 in the testes of DKO mice were significantly decreased compared with those in control mice (left column, Fig. 6A). The residual OSR1 expression in DKO mice could be explained by its presence in other cells of testicular tissues. We found that the expression of both total NKCC1 and p-NKCC1 (T206) was significantly reduced in the testicular tissues of DKO mice compared with those of control mice (right column, Fig. 6A). We next examined the cellular localization of NKCC1 and p-NKCC1 (T206) in the testicular tissue of DKO and control mice. In control adult mouse testes, co-immunostaining of NKCC1 or p-NKCC1 (T206) with vimentin showed that NKCC1 and p-NKCC1 are highly expressed in SCs, further supporting a role for this signalling pathway in SCs (Fig. 6B,C). Moreover, the signals and overall fluorescence intensity of NKCC1 and pNKCC1 were markedly attenuated in the seminiferous tubules of DKO mice (Fig. 6B,C), supporting the results obtained in western blot analysis. Collectively, these results indicate that an insufficiency of both OSR1 and SPAK in SCs resulted in impaired expression and phosphorylation of NKCC1 in testes, contributing to male infertility with impaired spermatogenesis. These results supported the notion that OSR1 and SPAK exerted mutually compensatory roles in regulating NKCC1 and affecting spermatogenesis.

## Discussion

To the best of our knowledge, this is the first report demonstrating that OSR1 and SPAK cooperatively modulate NKCC1 activity in SCs to support spermatogenesis. Our data indicate that functional NKCC1 in SCs, regulated by both OSR1 and SPAK, contributes to male fertility. NKCC1, a downstream target of OSR1 and SPAK, has been reported to play a pivotal role in spermatogenesis, as shown in NKCC1 KO mice^5^. NKCC1-dependent Cl^–^ currents are also essential for the zona pellucida-induced AR in sperm^6^. Although several *in vivo* studies have indicated that NKCCs are controlled by OSR1 and/or SPAK^12^,^13^,^24^,^36^, no study has examined how SPAK and OSR1—when co-expressed—might modulate NKCC1 activity in reproductive tissues and affect fertility. Our study provides direct evidence that male DKO mice with a deficiency of both kinases in their SCs, which leads to impaired phosphorylation of NKCC1, are infertile, demonstrating an essential role for OSR1 and SPAK in cooperatively controlling NKCC1 function and supporting spermatogenesis.

SPAK and OSR1 display high amino acid sequence homology in their N-terminal catalytic (96%) and C-terminal regulatory domains (67%)^37^, and both interact with NKCC1 through their CCT domain^9^,^38^, emphasizing the structural and functional similarities between these two kinases. The phylogenetic tree of the members of the GCK-VI subfamily of STE20p-related kinases illustrates that most non-mammalian species express only a single isoform with greater resemblance to OSR1 than SPAK, suggesting that SPAK has evolved from a gene duplication of OSR1 during the evolution of mammals^37^. Moreover, KO of the OSR1 orthologue, *Fray*, in *Drosophila* causes larval death^39^, similar to findings that global OSR1^*−*/*−*^, OSR1 gene-trapped, or kinase-dead knockin mice die during embryogenesis^13^,^36^,^37^. In contrast, global SPAK^*−*/*−*^, SPAK gene-trapped, or kinase-dead knockin mice were viable^12^,^36^,^37^, indicating a dominant role for OSR1 in the regulation of development. Furthermore, there was no reduction in the level of NKCC1 phosphorylation in testis and lung tissues from SPAK kinase-dead knockin mice^36^, suggesting that OSR1 principally phosphorylates this cotransporter and that SPAK can be dispensable in these tissues. Therefore, our observation of similar phenotypes of male fertility in SPAK^−/−^ and WT mice makes logical sense based on the evolutionary roles of these two genes, as indicated by the lethal phenotypes in *Fray*^−/−^ flies and OSR1^−/−^ mice, and the unaffected level of phosphorylated NKCC1 in the testes of SPAK-deficient mice. These phenotypes in SPAK^−/−^ mice also hint that other physiological compensatory effects—such as a parallel kinase like OSR1—are sufficient to overcome the loss of SPAK activity in either SCs or germ cells. Indeed, our data revealed upregulation of p-OSR1 in the absence of SPAK in testicular tissues of SPAK^−/−^ mice.

Subfertility was observed in male OSR1^+/−^ mice. One explanation for the fertility phenotype is that there is a developmental delay in OSR1^+/−^ males, but we observed no such developmental delay. In addition, OSR1^+/−^ mice grew normally and were indistinguishable from their WT littermates in appearance, sexual behaviour, and body weight. In the histological analysis, three of six OSR1^+/−^ mutants had a low level of cystic changes in their seminiferous tubules. These results may indicate incomplete phenotypic penetration in the OSR1^+/−^ mutant model, which would confound its interpretation.

Previously reported *in vitro* studies have revealed that NKCC is activated through a phosphorylation-dependent mechanism^40^,^41^ and that chloride is essential for capacitation-associated processes^6^. Furthermore, NKCC1 is likely to be responsible for the inward translocation of Cl^−^ in spermatozoa, and these NKCC-dependent Cl^−^ currents subsequently contribute to the zona pellucida-induced AR and fertilization. Taken together, these studies support the notion that phosphorylation of NKCC1 by OSR1 plays a role in fertilization. Therefore, OSR1 haploinsufficiency may cause decreased pNKCC1 abundance in testes and mature spermatozoa, thereby contributing to male subfertility.

SCs play an essential role in developing sperm cells at all stages of spermatogenesis^42^. They provide physical support, nutrients, and paracrine signals to all germ cell generations^43^. Because our data revealed that both OSR1 and SPAK are expressed in SCs, it was of interest to investigate their roles in these cells. However, loss of either OSR1 or SPAK in SCs did not affect male fertility. We also observed that p-OSR1 was upregulated in the absence of SPAK in adult testes from SPAK^−/−^ mice, implying that OSR1 may compensate for the loss of SPAK in testicular tissues. Indeed, ablation of both these kinases in SCs led to male sterility, highlighting their important roles in these cells. The co-expression and compensatory effects of OSR1 and SPAK in SCs suggests that these two kinases may act as key modulators with redundant homeostatic roles in prevention of an effect of the loss of either kinase on SC function.

NKCC1 is phosphorylated and activated by OSR1/SPAK^18^,^19^. Several lines of evidence also support the notion that OSR1 and SPAK are important regulators of NKCC1 *in vivo*^44^,^45^. These studies demonstrate that OSR1 and SPAK interact and stimulate NKCC1 activity by phosphorylating this cotransporter. Moreover, male NKCC1 KO mice are infertile because of defective spermatogenesis^5^. Given that NKCC1 is expressed in SCs and germ cells^5^, this raised the possibility that either this cotransporter plays a vital role in SC support of spermatogenesis or that it is directly required for the development and maturation of germ cells. Our data demonstrated that OSR1/p-OSR1, SPAK/p-SPAK, and NKCC1/p-NKCC1 were expressed in SCs. Ablation of both OSR1 and SPAK in SCs of DKO mice led to decreased NKCC1 function because of reduced NKCC1 abundance, and these mouse lines were infertile because of failed spermatogenesis. The impairment of SC functions in DKO mice may lead to the progressive loss of spermatogenic cells, suggesting a disturbed SC–germ cell interaction. These results are consistent with reports showing failure of spermatogenesis in mouse lines deficient in NKCC1^5^, supporting the important contribution of SC NKCC1 function to spermatogenesis.

In the rat, SCs and germ cells constitute 16% and 56% of the testicular volume, respectively^46^. When germ cells are depleted, a near-twofold increase (to 36%) in the proportion of the testicular volume constituted by SCs could be theoretically expected^29^. The expression of the SC-specific AR targets *Rhox5 (Pem*) and *Gata-1* have been determined in the homozygous mouse mutant dominant white spotting (*W*^*v*^/*W*^*v*^), a germ-cell-free model. The expression levels of *Gata-1* and *Rhox5* mRNA were twofold higher than those in the control mouse testes, probably reflecting the fact that the ratio of SCs to total testis cells was higher in *W*^*v*^/*W*^*v*^ mouse testes (without germ cells) than in the control mouse testes (with germ cells)^27^. Therefore, the near-twofold increase in the level of transcripts of the SC-specific markers *Sox9* and *Gata-1* in testicular tissues of DKO mice compared with control mice could be the result of the changes in the cellular composition of DKO testes. Moreover, the distribution of Gata-1- and Sox9-positive cells occupied almost all of the atrophic seminiferous tubules of DKO mice. Furthermore, there was no detectable expression of genes for selected differentiation markers (A-Myb, PABP, TP1, and protamine1) in the testes of DKO mice. Collectively, these data support the notion that the testes of DKO mouse were almost free of germ cells.

DKO testes displayed a SC-only phenotype, resulting from the changes in cellular composition. The cell types present in the DKO testes are substantially different from those observed in the control mice. As a result, relative mRNA levels cannot be compared across genotypes without known stable, cell-specific controls. The apparent phenomenon of different OSR1 abundance in testicular tissues of SC-OSR1^−/−^ and DKO mice probably derives from the different cellular composition of SC-OSR1^−/−^ (containing SCs, germ cells, and other cells) and DKO (containing SCs and other cells) testes. The apparent reduction in OSR1 in the DKO testes is probably related to the reduced number of germ cells. It is also possible that the reduction in OSR1, SPAK, and NKCC1 in the DKO testes is a result of the increased proportion of SCs because of the reduction in germ cells, and that the concurrent decrease in phosphorylation is a result of the reduction in total protein. To clarify further this important issue, we determined the expression of NKCC1 and p-NKCC1 in the testicular tissues of DKO mice. Double-IF staining for vimentin and NKCC1 or p-NKCC1 (T206) revealed marked attenuation of both NKCC1 and p-NKCC1 in the seminiferous tubules of DKO mice, supporting the results observed in western blot analysis.

In conclusion, our results demonstrate that OSR1 and SPAK are critical in the regulation of male fertility in SCs. Our data also indicate that OSR1 and SPAK cooperatively regulate male fertility by controlling the activity of NKCC1 in SCs. The markedly infertile phenotypes of the male DKO mice show that OSR1 and SPAK are potential biomarkers for the diagnosis and assessment of human male infertility.

## Material and Methods

### Mice

Mice were housed at the Animal Facility of the National Defense Medical Center, Taipei, Taiwan, under controlled environmental conditions. OSR1^+/−^, floxed *Oxsr1* (OSR1^F/F^), and SPAK^−/−^ mice were kindly provided by Professor Yang and were bred and genotyped as described^12^,^13^. C57BL/6 mice were purchased from the National Laboratory Animal Center, Taiwan. OSR1^+/−^ mice were intercrossed with C57BL/6 mice to generate OSR1^+/−^ and WT littermate mice. WT littermate mice were used as controls. Mice harbouring the anti-Müllerian hormone (AMH)-Cre transgene (stock number: 007915) with expression of Cre recombinase directed by the testis Sertoli cell (SC)-specific promoter elements of the *Amh* gene were purchased from The Jackson Laboratory (Bar Harbor, ME). SC-specific OSR1 KO mice (SC-OSR1^−/−^) were generated in this study by crossing pAMH-Cre transgenic mice^20^,^21^,^22^,^23^ and OSR1^F/F^ mice^13^. OSR1^F/F^ were crossed with AMH-Cre mice, and the resulting AMH-Cre(+)OSR1^F/+^ offspring were backcrossed to OSR1^F/F^ mice to obtain AMH-Cre(+)OSR1^F/F^, the so-called SC-OSR1^−/−^. Littermate mice that lacked the AMH-Cre transgene were used as controls. Generation of DKO mice that lacked both OSR1 and SPAK within SCs was performed as follows. SPAK^−/−^ mice were bred with AMH-Cre(+)OSR1^F/F^ (SC-OSR1^−/−^) mice, and the resulting AMH-Cre(+)OSR1^F/+^.SPAK^+/−^ and AMH-Cre(–)OSR1^F/+^.SPAK^+/−^ offspring were intercrossed to obtain homozygous mice with invalid OSR1 alleles (OSR1^F/F^), which were intercrossed to obtain males with key genotypes: AMH-Cre(+)OSR1^F/F^.SPAK^−/−^(SC-OSR1^−/−^.SPAK^−/−^ [DKO]) and their respective contemporary littermate controls: AMH-Cre(–)OSR1^F/F^.SPAK^+/+^. All animal experiments were conducted in accordance with institutional guidelines and were approved by the National Defense Medical Center Institutional Animal Care and Use Committee.

### Mouse genotyping

Mouse genomic DNA was isolated by SDS/proteinase K digestion and phenol/chloroform extraction as previously described^47^. PCR genotyping of Cre, OSR1, and SPAK alleles has been described previously^12^,^13^,^48^. To verify that excision was restricted to the testes, DNA was extracted from various tissues, including testes from adult mice at 5 months, and PCR was performed using the primers previously described^13^ to detect the deleted allele lacking exon 9 and 10 (OSR1 [Δexon 9, 10]): forward: 5′-AAA-CCT-GCT-GGG-CTT-CTA-TG-3′; reverse 5′-TGG-TGA-AAT-GGC-AAA-TGT-GT-3′. The annealing temperature was 58.5 °C. The PCR products were analysed either by agarose gel or using the LabChip GX (Caliper Life Sciences, Hopkinton, MS).

### Fertility assessment

Age-matched WT littermates, OSR1^+/−^, and age-matched SPAK^−/−^ (8–24 weeks of age); SC-OSR1^−/−^ and control (8 weeks of age); and DKO and control (8 weeks of age) males were housed singly with a fertile WT female (>6 weeks of age) for a period of 2 months^49^. The timing of delivery and the number of offspring born were recorded.

### Tissue collection and histology

After male mice were euthanized by cervical dislocation or CO_2_ inhalation, their body weights were determined. Testes and epididymides were dissected free of fat and connective tissue and were weighed separately. Tissues were fixed in either Bouin’s solution or 10% neutral buffered formalin, embedded in paraffin, sectioned (5 μm), and stained with haematoxylin and eosin.

### Sperm assessment

The caudal regions of the epididymides were removed from male mice that had been euthanized by cervical dislocation or CO_2_ inhalation. The caudal epididymis was minced in 1 mL of human tubal fluid (HTF) medium (Irvine Scientific, Santa Ana, CA), containing 3 mg/mL of BSA, pre-equilibrated at 37 °C, 5% CO_2_, and incubated for 20 min to allow spermatozoa to leave the epididymis. Spermatozoa were counted in a Makler counting chamber (Sefi Medical Instruments, Haifa, Israel) after a 1/6 dilution. Sperm mobility was assessed by manual methods. The motility of each spermatozoon was graded as progressive motility, non-progressive motility, or immotility according to the criteria of the 5th Edition of the World Health Organization (WHO) Laboratory Manual for the Examination and Processing of Human Semen^50^.

Spermatozoa suspended in HTF medium containing 3 mg/mL of BSA were spread over clean glass slides and air-dried. The slides were fixed and stained using a Diff-Quik Stain Kit (BDH, Poole, UK) according to the manufacturer’s instructions. We first photographed spermatozoa under light microscopy at ×400 magnification, and the morphology of the spermatozoa was later examined using a personal computer program. At least 200 spermatozoa were observed. Sperm morphology was categorized as normal or abnormal. Abnormal spermatozoa were further classified as spermatozoa with an abnormal head only, spermatozoa with an abnormal tail only and spermatozoa with abnormal head and abnormal tail^51^,^52^,^53^. All preparations for analysis of sperm morphology were coded and scored blind.

### *In vitro* fertilization (IVF)

The IVF assay was performed as previously described with some modifications^49^,^53^. Mature WT female mice (6–12 weeks old) were superovulated by injection with 7 IU pregnant mare’s serum gonadotropin (Sigma-Aldrich, Seoul, South Korea), and 48 h later with 7 IU human chorionic gonadotropin (Sigma-Aldrich). After 16–18 h, the oocyte-cumulus complexes (OCCs) were collected from the oviducts and pooled in 200-μL droplets of HTF medium containing 3 mg/mL bovine serum albumin (BSA) (Sigma-Aldrich) under mineral oil (Sigma-Aldrich) (20–30 oocytes/well). Fresh clots of cauda epididymal spermatozoa were prepared and incubated for 20 min in 100-μL droplets of HTF medium containing 3 mg/ml BSA under mineral oil to allow spermatozoa swim out, and were allowed to capacitate for 60 min in 5% CO_2_ at 37 °C. Then, 300,000 sperm^49^ were added to each well containing OCCs and incubated for a further 6 h. After a series of washes with two drops of HTF medium containing 3 mg/mL BSA using a thin bore pipette, eggs were removed to 100-μL droplets of KSOM medium (Millipore, Billerica, MD) under mineral oil for 18 h at 37 °C. Thereafter, the oocytes were assessed and classified as non-fertilized oocytes (had not divided), fertilized oocytes (divided), or dead oocytes^53^. The percentage of fertilization was determined for each fertilization drop by dividing the number of fertilized oocytes by the total number of oocytes (sum of fertilized and non-fertilized oocytes) in that drop and multiplying this ratio by 100.

### Immunoblotting and immunofluorescence staining

Semi-quantitative immunoblotting (IB) and IF microscopy were performed as previously described^12^,^13^,^54^ to assess relative expression levels of proteins of interest using whole homogenate without nuclear fraction (6000 × *g*) or crude membrane fraction (17,000 × *g*) from TM4 cells, testes, and spermatozoa (at least two mice). The whole homogenate without nuclear fraction was used for measurement of OSR1/p-OSR1 and SPAK/p-SPAK by sodium dodecyl sulfate-polyacrylamide gel electrophoresis (SDS-PAGE) and IB. The crude membrane fraction was used to detect NKCC1/p-NKCC1 by SDS-PAGE and IB. Protein concentrations were measured using the BCA kit from Pierce. In each lane, 100 μg and 50 μg of total protein from testes and spermatozoa, respectively, were loaded on an SDS-8% PAGE and transferred onto polyvinylidene difluoride membranes. After blocking nonspecific binding with 3% BSA (Sigma-Aldrich) in Tris-buffered saline with 0.05% Tween 20, the blots were incubated with specific antibodies. An antibody that could recognize both p-OSR1 (S325) and p-SPAK (S383) simultaneously (1:2000)^13^,^44^,^55^, an antibody that could recognize both OSR1 and SPAK simultaneously (1:2000)^45^,^55^, and a rabbit anti-p-NKCC2 (T96) antibody, which could also recognize p-NKCC1 (T206) (1:2000)^12^,^13^, were used for IB and were kindly provided by Professor Yang. Other commercially available primary antibodies were used for IB, including NKCC (T4)^13^,^56^ and anti-β-actin (1:10,000) (AC-15; Sigma-Aldrich). The secondary antibodies used for IB (1:3000) were alkaline phosphatase-conjugated anti-IgG antibodies (Promega, Madison, WI), and the signals were detected with Western Blue reagent (Promega). The intensity of bands was assessed using Image J (NIH, Bethesda, MD). The primary antibodies used in IF were either rabbit polyclonal anti-OSR1 (1:100) (Cell Signaling Technology, Danvers, MA), rabbit polyclonal anti-SPAK (1:100) (Cell Signaling), mouse monoclonal anti-vimentin (1:500) (Abcam, Cambridge, UK), goat polyclonal anti-GATA-1 (1:200) (Santa Cruz Biotechnology, Dallas TX), rabbit polyclonal anti-SOX9 (1:200) (Millipore), anti-NKCC (T4)^13^,^56^, or anti-p-NKCC2 (T96) (1:200), which could also recognize p-NKCC1 (T206) (1:200)^12^,^13^. Alexa-488 or -566 dye-labelled (Molecular Probes, Eugene OR) secondary antibodies were used for IF microscopy. IF images were photographed using a Leica DM2500 fluorescence microscope.

### RT-PCR and RT-qPCR

Total RNA was extracted (Qiagen, Valencia, LA, CA, USA) and used for cDNA synthesis with the SuperScript III synthesis kit (Invitrogen, Carlsbad, CA, USA) according to the manufacturer’s instructions. The relative expression of each mRNA was normalized to that of *Rps2*. All experiments were performed three times in duplicate. The primers used for RT-qPCR using the SYBR Green method are shown in Table S1 in the supplementary information.

### Statistical analysis

All results were expressed as mean ± S.E.M. The log-rank (Mantel–Cox) test was used for comparison of the time to first litter. ANOVA and the Tukey post hoc test were used to compare the differences among the three or four groups. The Student *t* test was applied for statistical analysis of other experiments in this study. A *p* value less than 0.05 was considered to be significant.

## Additional Information

**How to cite this article**: Liu, Y.-L. *et al.* OSR1 and SPAK cooperatively modulate Sertoli cell support of mouse spermatogenesis. *Sci. Rep.* **6**, 37205; doi: 10.1038/srep37205 (2016).

**Publisher’s note**: Springer Nature remains neutral with regard to jurisdictional claims in published maps and institutional affiliations.

## Supplementary Material

Supplementary Information
