# Supplementary material for: OSR1 and SPAK cooperatively modulate Sertoli cell support of mouse spermatogenesis
Source: Sci Rep. 2016 Nov 17;6:37205. doi: 10.1038/srep37205 (PMC5112561; doi:10.1038/srep37205)
Supplement: Supplementary Information [file srep37205-s1.doc]

# OSR1 and SPAK cooperatively modulate Sertoli cell support of mouse spermatogenesis

Yung-Liang Liu^1,2,3^, Sung-Sen Yang^1,4^, Shyi-Jou Chen^5,6^, Yu-Chun Lin^1,7^, Chin-Chen Chu^8,9^, Hsin-Hui Huang^2^, Fung-Wei Chang^2^, Mu-Hsien Yu^2^, Shih-Hua Lin^1,4^, Gwo-Jang Wu^1,2,*^, Huey-Kang Sytwu^1,5,10,*^

1 Graduate Institute of Medical Sciences, National Defense Medical Center, Taipei 114, Taiwan

2 Department of Obstetrics and Gynecology, Tri-Service General Hospital, National Defense Medical Center, Taipei 114, Taiwan

3 Department of Obstetrics and Gynecology, Tri-Service General Hospital Penghu Branch, Penghu 880, Taiwan

4 Division of Nephrology, Department of Medicine, Tri-Service General Hospital, National Defense Medical Center, Taipei 114, Taiwan

5 Department and Graduate Institute of Microbiology and Immunology, National Defense Medical Center, Taipei 114, Taiwan

6 Department of Pediatrics, Tri-Service General Hospital, National Defense Medical Center, Taipei 114, Taiwan

7 Department of Pathology, Tri-Service General Hospital, National Defense Medical Center, Taipei 114, Taiwan

8 Department of Anesthesiology, Chi Mei Medical Center, Tainan, Taiwan
9 Department of Recreation and Health-Care Management, Chia Nan University of Pharmacy and Science, Tainan, Taiwan

10 Graduate Institute of Life Sciences, National Defense Medical Center, Taipei 114, Taiwan

*Corresponding author. E-mail: sytwu@ndmctsgh.edu.tw

[Supplementary Information](http://embor.embopress.org/authorguide#suppinf1)

# SI Material and methods

## Serum testosterone assay

The blood of male mice was drawn by cardiocentesis and incubated at room temperature for 2 h. After centrifugation at 2000 × *g* for 20 min, the serum was collected and stored at –80 °C until analysis. The levels of serum testosterone were measured using a testosterone enzyme immunoassay kit (Cayman Chemical Company, Ann Arbor, MI, USA).

## Acrosome reaction

AR was performed as previously described^1,2^. Briefly, after 1 h capacitation, spermatozoa were treated with calcium ionophore (A23187; 10 μM) (Sigma-Aldrich) for an additional 30 min to induce AR and were then smeared on microscope slides at room temperature and allowed to dry. After air-drying, smears of spermatozoa were fixed in cold absolute methanol for 15 min, washed once in phosphate-buffered saline (PBS) and twice in distilled water at 5-min intervals, air-dried, incubated with fluorescein isothiocyanate-conjugated peanut agglutinin (Vector Laboratories) in PBS for 30 min, and finally washed three times with PBS. We first photographed spermatozoa using a fluorescence microscope at ×400 magnification. AR was subsequently assessed using a personal computer program. Spermatozoa with equatorial green staining or no staining at all were considered to be acrosome reacted, and cells with green staining over the acrosomal cap were considered to be acrosome intact^2^. At least 200 spermatozoa were counted per slide. All preparations for analysis of AR were coded and scored blind. The percentage of acrosome-reacted spermatozoa was determined in each experimental condition by dividing the number of acrosome-reacted spermatozoa by the total number of spermatozoa scored (sum of acrosome-reacted and non-acrosome-reacted) and multiplying this ratio by 100^3^. Positive (stimulation with ionophore) and negative (no stimulation) controls were included in all experiments.

## Effect of an NKCC inhibitor on fertilization

Fresh clots of cauda epididymal spermatozoa from wild-type (WT) and OSR1^+/–^ mice were prepared and incubated for 20 min in 200-μL droplets of human tubal fluid (HTF) medium without bovine serum albumin (BSA) under mineral oil to allow spermatozoa to swim out. Because the furosemide was dissolved in dimethyl sulfoxide (DMSO), 5% DMSO was present under all conditions tested to control for deleterious solvent effects on fertilization. Fifty microliter droplets of HTF medium containing spermatozoa were added to 50-μL droplets containing DMSO or 2 mM furosemide in DMSO in HTF with 6 mg/ml BSA and incubated for 60 min in 5% CO_2_ at 37 °C. Afterward, the spermatozoa were washed and centrifuged for 2 min at 800 × *g*, and the pellet was resuspended in 50-μL droplets of HTF medium containing 3 mg/mL BSA under mineral oil; 300,000 sperm^1^ were added to the wells containing oocyte-cumulus complexes (OCCs). The following *in vitro* fertilization (IVF) procedures were as described in the Methods section.

## Terminal deoxynucleotidyl transferase dUTP nick end labelling (TUNEL) assay

TUNEL staining was used to detect apoptosis using an *in situ* cell death detection kit according to manufacturer’s instructions (Roche, Indianapolis, IN, USA). Images were captured on a Leica DM2500 fluorescence microscope. Four high-power fields were selected for analysis of each stain. Image analysis was performed using Image J (NIH, Bethesda, MD).


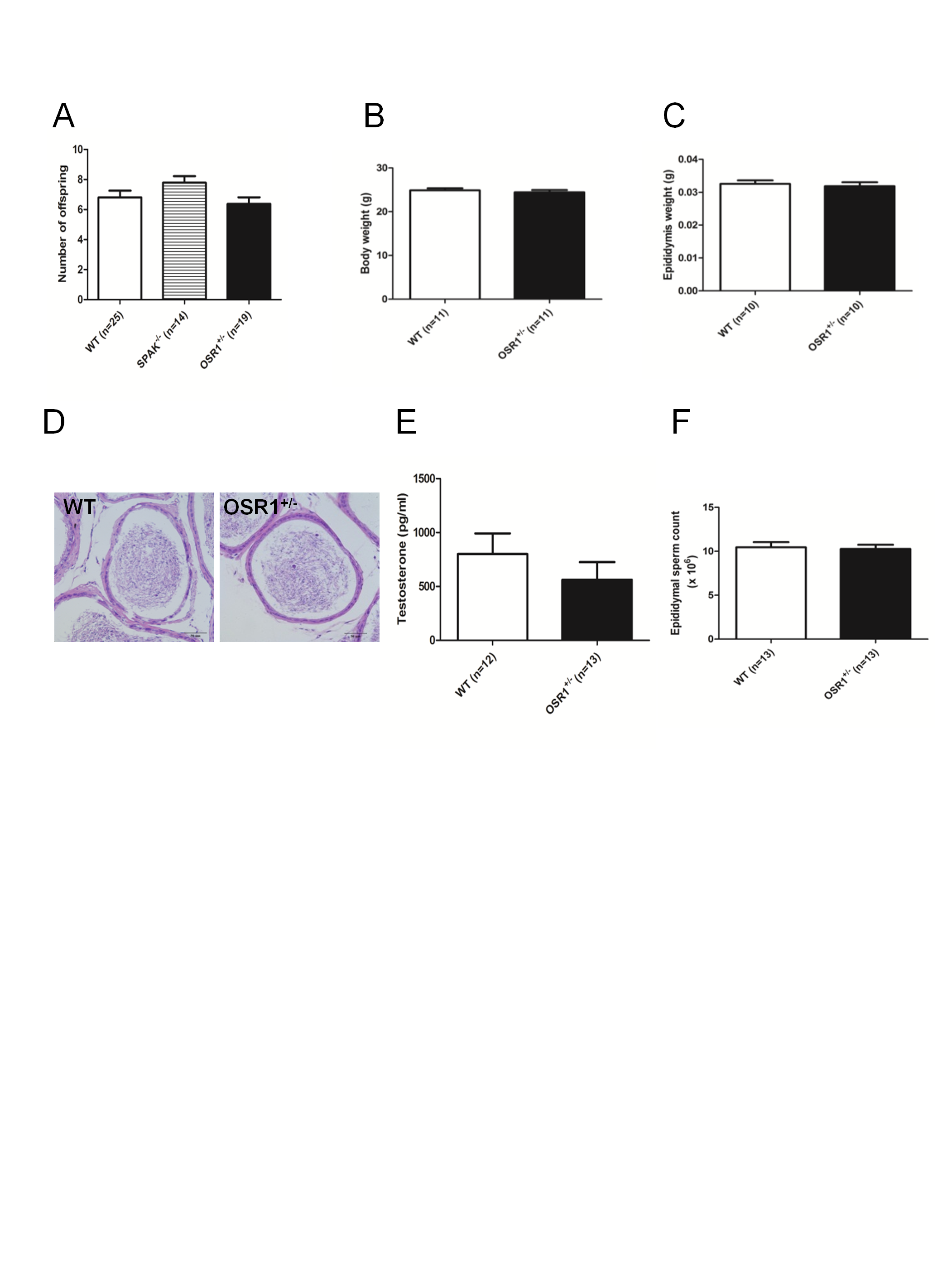


Figure S1. Phenotypic assessment, sperm assessment, and serum testosterone levels.

(A) The average litter size. Comparison between the average litter size generated by WT, SPAK^–/–^, or OSR1^+/–^ males mated with WT females. Each bar represents the mean ± SEM. *p* > 0.05 by one-way analysis of variance. (B) Mouse body weight. (C) Epididymis weight. (D) Histological analysis of epididymis sections. Scale bar: 50 μm. (E) Serum testosterone levels. (F) Epididymal sperm count. Spermatozoa were isolated from cauda epididymal tissues and counted. All values in A, B, C, F, and G are given as the mean ± SEM. *p* > 0.05 by two-tailed Student’s unpaired *t* test.

**
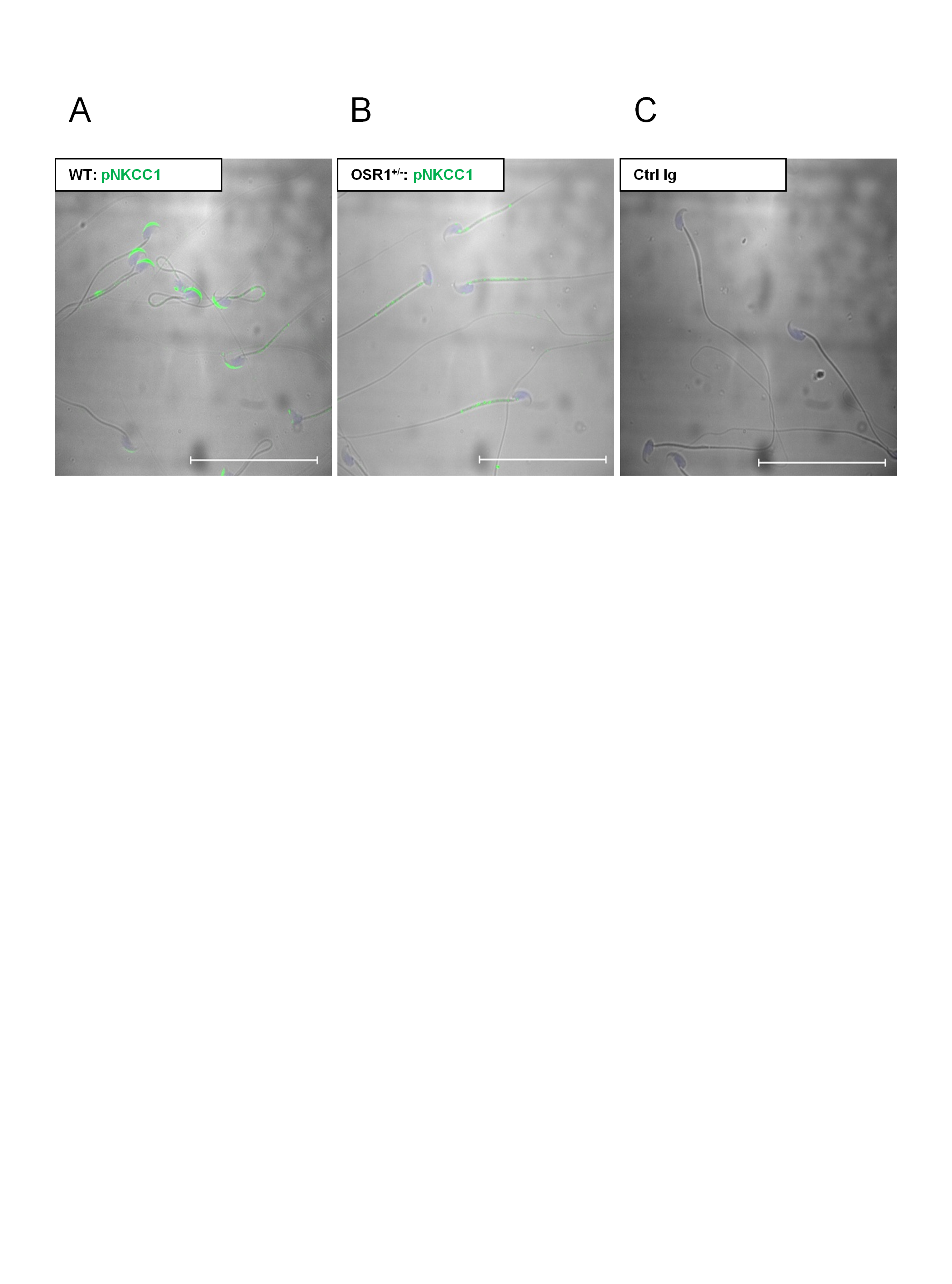
**

Figure S2. p-NKCC1 expression in epididymal spermatozoa.

Representative immunofluorescence (IF) staining of p-NKCC1 (green) in spermatozoa of WT (A) and OSR1^+/–^ (B) mice. (C) Control staining with nonspecific immunoglobulin. The signal was markedly reduced in OSR1^+/–^ spermatozoa. For IF assays, mouse spermatozoa were air-dried, fixed, permeabilized, and stained with anti-p-NKCC2 (T96) antibodies. Each experiment was repeated at least three times. Scale bar: 50 μm.


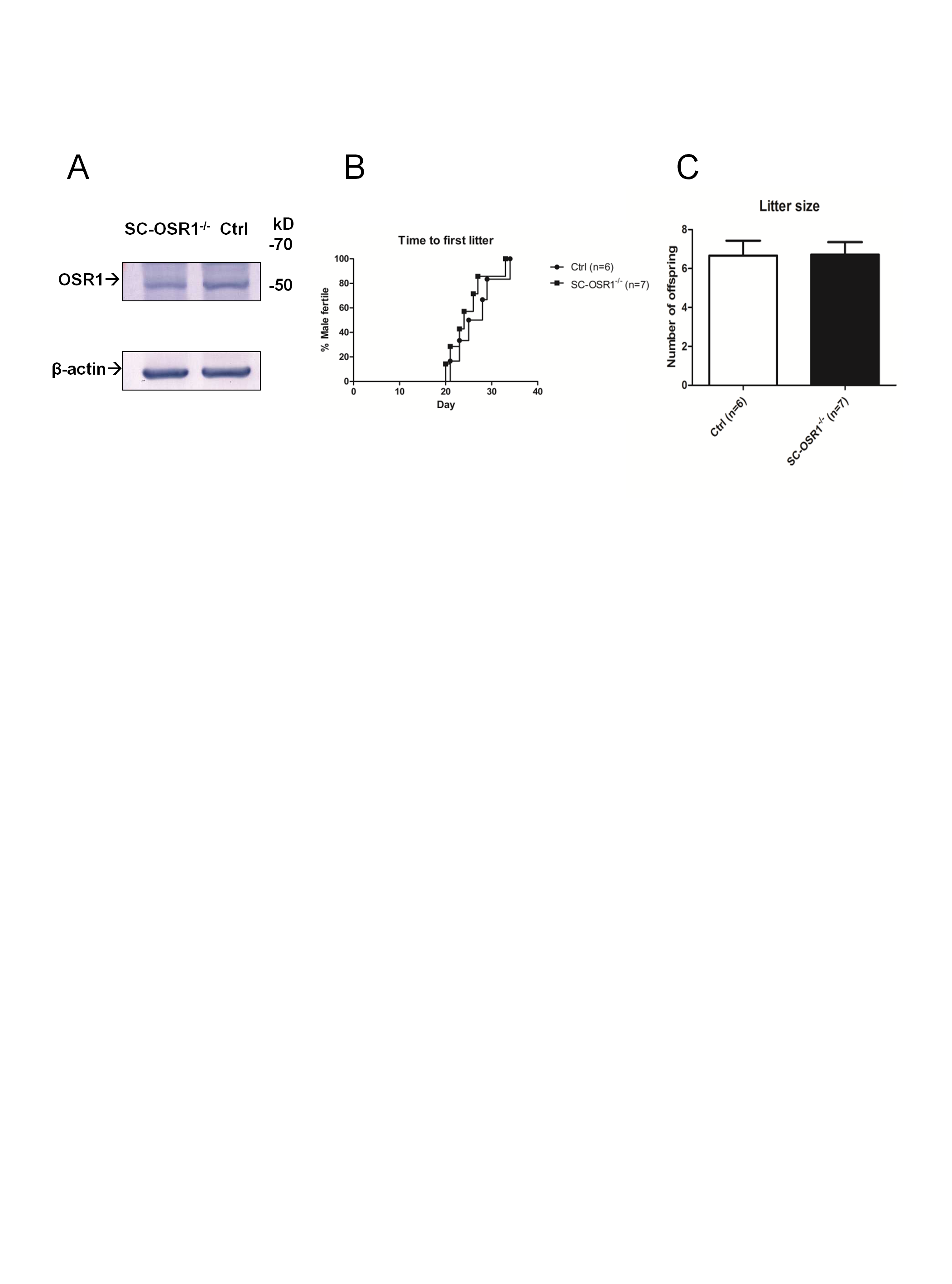


Figure S3. Generation of SC-OSR1^–/–^ mice and fertility assessment.

(A) Attenuation of OSR1 expression in SC-OSR1^–/–^ testes. The cell extracts of testes from SC-OSR1^–/–^ and control mice were analysed by sodium dodecyl sulfate polyacrylamide gel electrophoresis and Western blotting with the indicated antibodies. Data are representative of at least three experiments. β-Actin was used as a loading control. (B) Time to first litter. One male was crossed with one WT female. Fertility was confirmed when the male was able to sire offspring. *p* > 0.05 by log-rank test. (C) The average litter size. Comparison between the average litter size generated by SC-OSR1^–/–^ or WT males mated with WT females. Each bar represents the mean ± SEM. *p* > 0.05 by two-tailed Student’s unpaired *t* test.


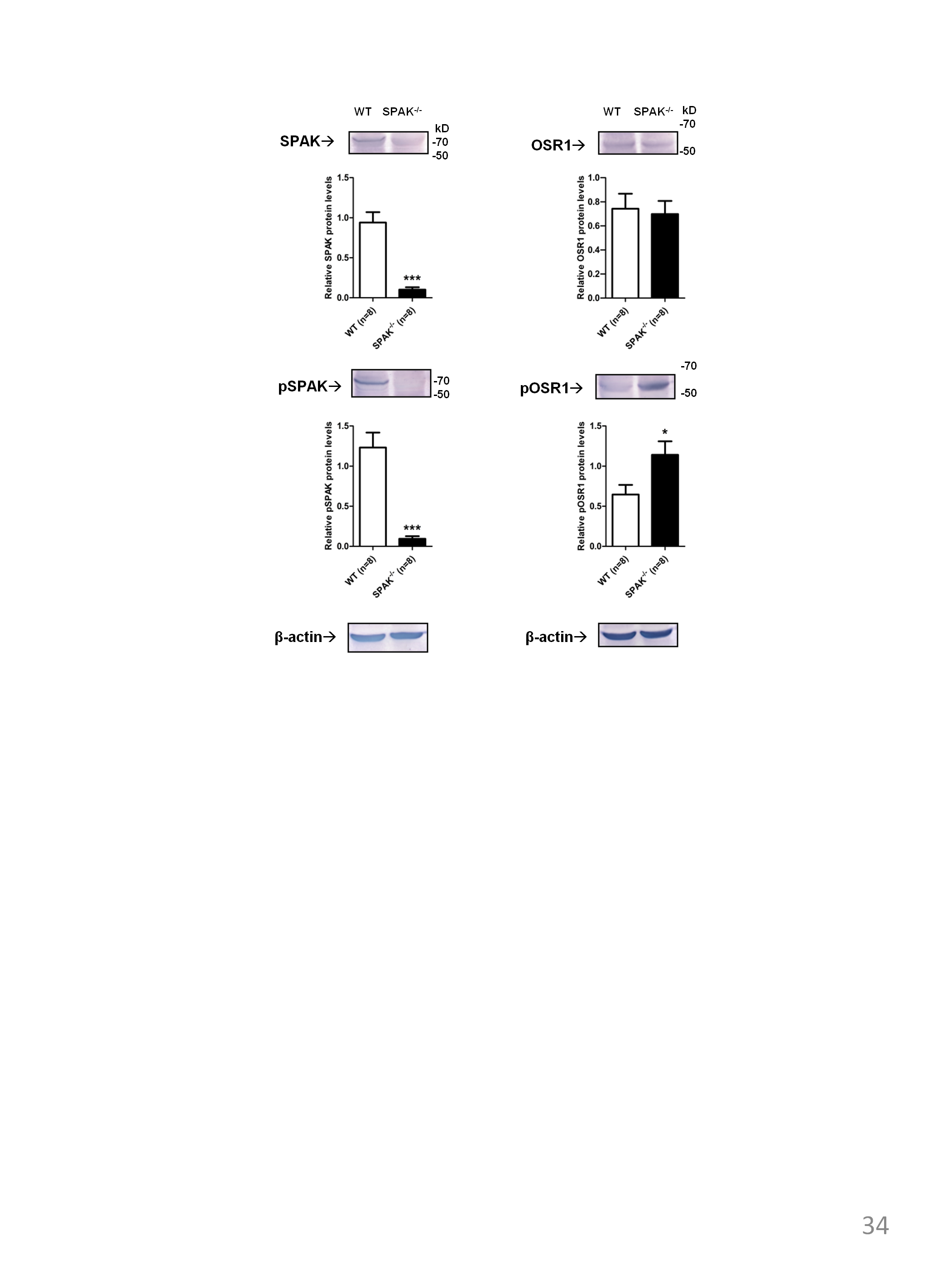


Figure S4. Upregulation of p-OSR1 in the absence of SPAK in testicular tissues of SPAK^–/–^ mice.

SPAK^–/–^ mice have reduced total and p-SPAK (left panel) but increased p-OSR1 (right panel) expression in testicular tissues. Semi-quantitative immunoblotting (upper) and densitometry (lower) of total and p-OSR1, and total and p-SPAK in testicular tissues of WT and SPAK^–/–^ mice at 5 months of age.


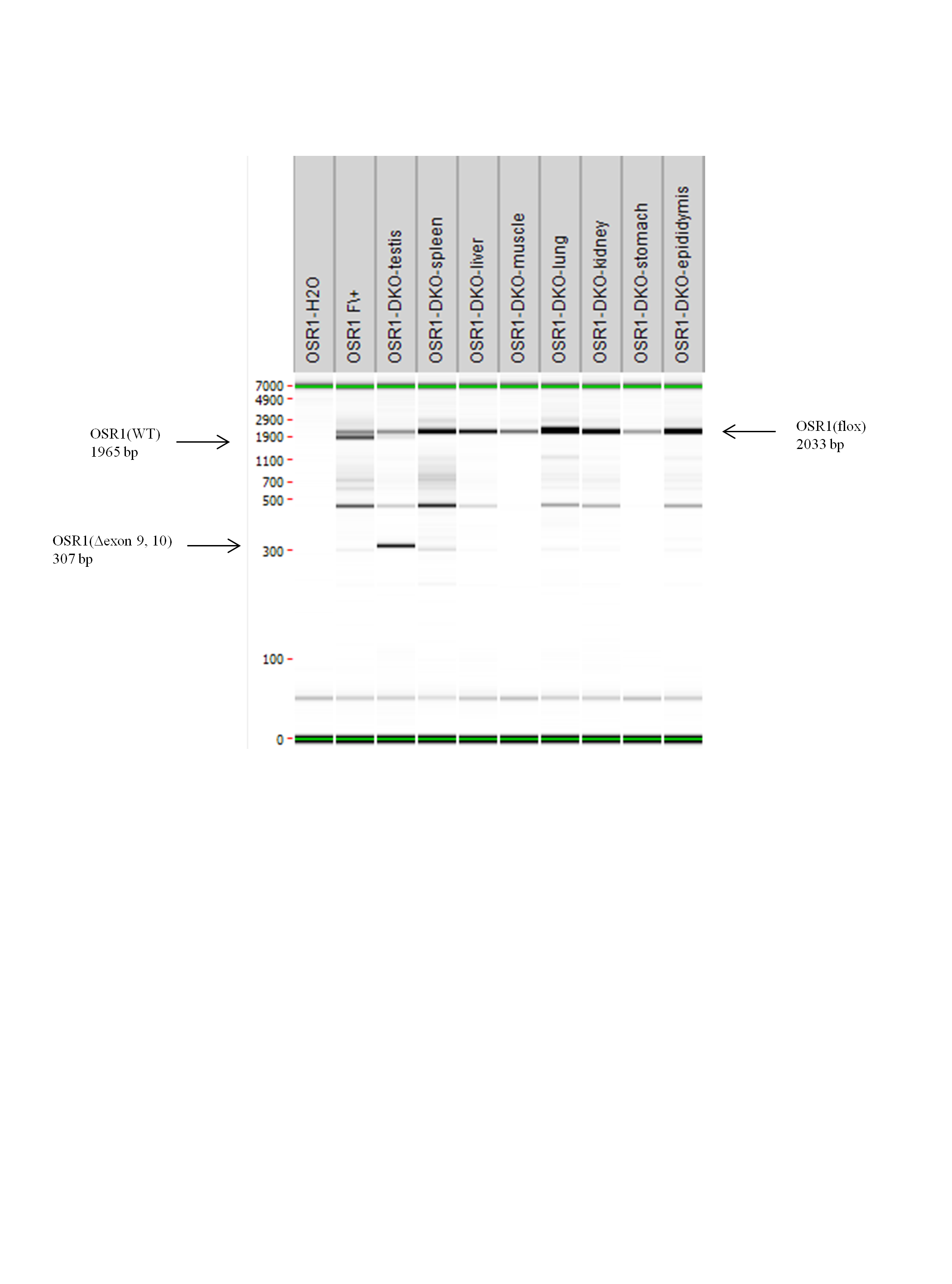


Figure S5. Detection of deleted (floxed) OSR1 allele in DKO testes using PCR*.*

In testes from adult DKO males at 5 months of age, specific primers amplified the 307 bp fragment corresponding to the deleted (floxed) OSR1 allele (OSR1[Δexon 9, 10]: deletion of exon 9 and 10 leading to a null allele of OSR1), which was not detected in other tissues. Genomic DNA derived from the tail of OSR1^F/+^ mice was used as a control. The upper fragment (2033 bp) represents the intact floxed allele, whereas the 1965-kb band represents the WT allele^4^.


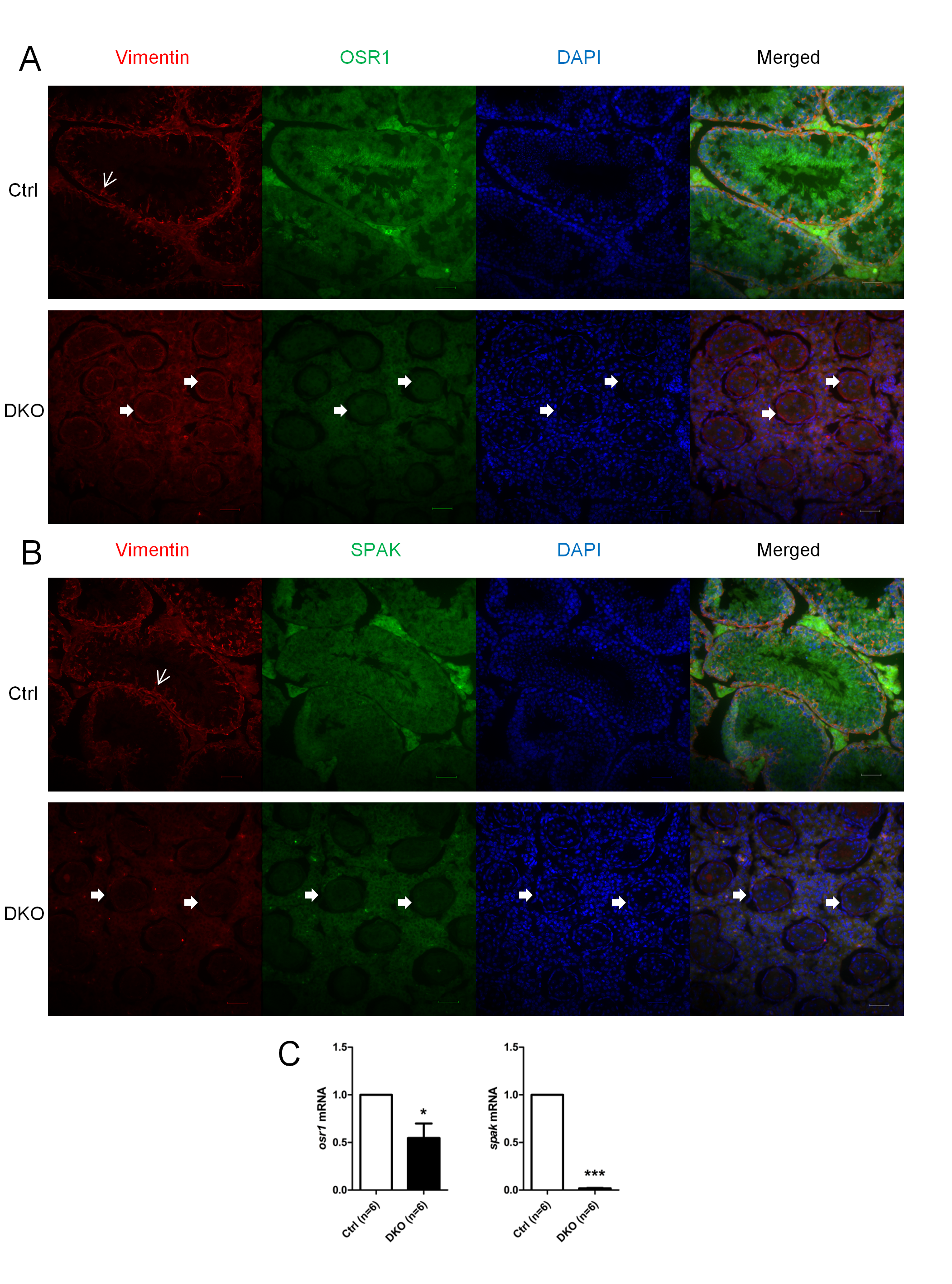


Figure S6. OSR1 and SPAK expression in testicular tissues of DKO mice.

(A) OSR1 expression in adult testes of control (upper panel, green) and DKO (lower panel, green) mice at 5 months of age. Vimentin (red) was expressed in the SC cytoplasm, and characteristic apical filament projections (arrow) were observed in adult testes of control mice. Double immunostaining for OSR1 and vimentin revealed that OSR1 was co-localized with vimentin in SCs. The diameters of seminiferous tubules (lower panel, arrow) of DKO testes were smaller than those of control mice. The signals for OSR1 were markedly reduced in the seminiferous tubules (lower panel, arrow) of DKO mice compared with controls. (B) SPAK expression in adult testes of control (upper panel, green) and DKO (lower panel, green) mice at 5 months of age. Vimentin (red) was expressed in the SC cytoplasm, and characteristic apical filament projections (arrow) were observed in adult testes of control mice. Double immunostaining for SPAK and vimentin revealed that SPAK was co-localized with vimentin in SCs. The diameters of seminiferous tubules (lower panel, arrow) of DKO testes were smaller than those of control mice. The signals for SPAK were markedly reduced in the seminiferous tubules (lower panel, arrow) of DKO mice compared with controls. Nuclei were counterstained with DAPI (blue). Scale bar: 50 μm. (C) Expression levels of *Oxsr1* and *Spak* genes in testicular tissues of DKO mice at 5 months of age. Both *Oxsr1* and *Spak* transcripts were significantly downregulated in testicular tissues from DKO mice compared with controls.

Table S1 Primers used for SYBG-based qPCR assays.

| .Gene | Forward primer | Reverse primer |
| --- | --- | --- |
| *A-myb ^5^* | GGTCTTCATCAAAACTTCAACACAA | TGAACAGGAATGTAAAACTGATTCTG |
| *PABP ^5^* | CCTTCATCAGCCCCTTGCT | TGGGATCCTCGCCTGGT |
| *TP1 ^5^* | GCAAGAACCGAGCTCCTCAC | GGACGCTCTTCCGGTATTTTC |
| *Protamin1 ^5^* | CAGCAAAAGCAGGAGCAGATG | GGCGACGGCAGCATCTTC |
| *Gata-1 ^6^* | TCCTCTGCATCAACAAGCCCA | GTTGAGCAGTGGATACACCTG |
| *Sox9 ^7^* | AGTACCCGCATCTGCACAAC | TACTTGTAATCGGGGTGGTCT |
| *Vimentin ^8^* | GCACCCTGCAGTCATTCAGA | GCAAGGATTCCACTTTCCGTT |
| *Rps2 ^9^* | ctgactcccgacctctggaaa | gagcctgggtcctctgaaca |
| *Osr1* | AAgATAAAgAAATgCTgAAgAAATATgg | AgTTCTgCTgCTgTTgg |
| *Spak* | CAggTgAgAggCTATgACT | gCACTTTCATTggAgggTAT |

# References

1 Simanainen, U., McNamara, K., Davey, R. A., Zajac, J. D. & Handelsman, D. J. Severe subfertility in mice with androgen receptor inactivation in sex accessory organs but not in testis. *Endocrinology* **149**, 3330-3338, doi:10.1210/en.2007-1805 (2008).

2 Ganaiem, M. *et al.* Effect of interleukin-1 receptor antagonist gene deletion on male mouse fertility. *Endocrinology* **150**, 295-303, doi:10.1210/en.2008-0848 (2009).

3 Wertheimer, E. V. *et al.* Chloride Is essential for capacitation and for the capacitation-associated increase in tyrosine phosphorylation. *J Biol Chem* **283**, 35539-35550, doi:10.1074/jbc.M804586200 (2008).

4 Lin, S. H. *et al.* Impaired phosphorylation of Na+-K+-2Cl- cotransporter by oxidative stress-responsive kinase-1 deficiency manifests hypotension and Bartter-like syndrome. *Proc Natl Acad Sci U S A*, doi:10.1073/pnas.1107452108 (2011).

5 De Gendt, K. *et al.* A Sertoli cell-selective knockout of the androgen receptor causes spermatogenic arrest in meiosis. *Proc Natl Acad Sci U S A* **101**, 1327-1332, doi:10.1073/pnas.0308114100 (2004).

6 Lindeboom, F. *et al.* A tissue-specific knockout reveals that Gata1 is not essential for Sertoli cell function in the mouse. *Nucleic Acids Res* **31**, 5405-5412 (2003).

7 Wainwright, E. N. *et al.* SOX9 regulates microRNA miR-202-5p/3p expression during mouse testis differentiation. *Biol Reprod* **89**, 34, doi:10.1095/biolreprod.113.110155 (2013).

8 Boor, P. *et al.* Complement C5 mediates experimental tubulointerstitial fibrosis. *J Am Soc Nephrol* **18**, 1508-1515, doi:10.1681/asn.2006121343 (2007).

9 Chang, Y. F., Lee-Chang, J. S., Panneerdoss, S., MacLean, J. A., 2nd & Rao, M. K. Isolation of Sertoli, Leydig, and spermatogenic cells from the mouse testis. *BioTechniques* **51**, 341-342, 344, doi:10.2144/000113764 (2011).
